# Integrated treatment program for alcohol related problems in community hospitals, Songkhla province of Thailand: A social return on investment analysis

**DOI:** 10.1371/journal.pone.0209210

**Published:** 2019-01-02

**Authors:** Athip Tanaree, Sawitri Assanangkornchai, Wanrudee Isaranuwatchai, Kednapa Thavorn, Peter C. Coyte

**Affiliations:** 1 Epidemiology Unit, Faculty of Medicine, Prince of Songkla University, Hat Yai, Songkhla, Thailand; 2 Institute of Health Policy, Management and Evaluation, University of Toronto, Toronto, Canada; 3 Ottawa Hospital Research Institute, the Ottawa Hospital, Ottawa, Canada; Stellenbosch University, SOUTH AFRICA

## Abstract

**Objectives:**

To estimate the impacts and social value relative to the cost of the Integrated Management of Alcohol Intervention Program in the Health Care System (i-MAP) on direct beneficiaries, using a Social Return on Investment (SROI) analysis.

**Method:**

A mixed-method approach was conducted among stakeholders and 113 drinkers (29 low-risk, 43 high-risk, and 41 dependent drinkers) who consecutively received i-MAP at four community hospitals in Songkhla province of Thailand. Resources for program implementation as well as drinking and a list of psychosocial outcomes, selected through stakeholder interviews, were measured among participants during and at the sixth month after participation, respectively. SROI (societal benefit-to-cost) ratio of i-MAP was estimated over a 5-year time horizon and shown in 2017 Thai baht, where US$1.00 = 33.1 baht. One-way and probabilistic sensitivity analyses of key parameters were performed among treatment subgroups.

**Results:**

Baseline estimates of the annual cost and 5-year social value of i-MAP were 25.5 and 51.0 million baht, respectively, yielding an estimated SROI ratio of 2.0, with a possible range of 1.3 to 2.4. Value created by the program was mostly attributed to broader gains to society (productivity gains and averted crime costs) and drinkers. Subgroup analyses suggested that the SROI ratio for high-risk drinkers was twice that for dependent drinkers (2.8 vs. 1.5). The probabilistic sensitivity analysis showed that more than 99% of the simulated treatments for both high-risk and dependent groups yielded benefits beyond the corresponding costs.

**Conclusions:**

By considering societal perspective, the i-MAP program has demonstrated its social value is twice its investment cost and potential for the program to be implemented nationwide.

## Introduction

Society suffers from alcohol-related diseases, injuries and mortality [1–3] which account for a substantial burden of disease and economic losses particularly among middle-income countries [3]. In Thailand, the social cost of alcohol consumption was estimated at 156 billion baht in 2006 (US$5 billion), representing 2% of the gross domestic product [4]. This could be an underestimation as plenty of intangible costs, such as societal concerns, domestic violence, caregiver stress and psychological suffering even among problem drinkers themselves [5–9], were not explicitly measured.

Accessible and affordable treatment programs for alcohol control in the community are recommended and cost-effective measures [10] for addressing these problems through a holistic and supportive environment [11]. In Thailand, the Integrated Management of Alcohol Intervention Programs in the Health Care System (i-MAP) was initiated by the Thai Health Promotion Foundation (Thaihealth) in 2011 to provide a comprehensive care process targeting different levels of alcohol users who receive general healthcare services. The project has provided guidelines and training for screening, brief and intensive interventions, detoxification and aftercare to healthcare personnel at different levels. At present, 60 community (district-level) hospitals across Thailand have integrated the program into usual care. According to the project report, healthcare personnel were generally satisfied with i-MAP and found it improved their confidence in managing those complicated cases. However, due to everyone’s workload, questions regarding the feasibility and worthiness of fully devoting their time to the program were also pointed out [12].

A range of alcohol interventions from brief to intensive, with or without conjunctive pharmacotherapies, were shown to be cost-effective as compared to usual treatment [13–20]. Units of measurement of effectiveness mostly include clinical outcomes (e.g. drinking patterns) and well-being (e.g. quality-adjusted life year; QALY). Notwithstanding the evidence, challenges arise when it comes to communicating the clinical results to the public. How their money could help reduce the intensity of drinking or gain certain QALYs, although important, may not be the only factors considered by the public funders. Furthermore, cost-benefit studies that presented more comprehensible results through monetization may still underestimate the true value of alcohol interventions as they only captured clinically relevant outcomes (e.g. healthcare cost saving) [17, 21]. Previous studies demonstrated that psychosocial outcomes of alcohol treatments may not be totally accounted for reduced alcohol consumption as they persist regardless of post-treatment drinking status. Hence any treatment of alcohol use should be assessed more comprehensively [22, 23].

Apart from traditional economic analyses, a recently developed Social Return on Investment (SROI) analysis has the advantage of measuring broader socio-economic outcomes. According to SROI methodology, psychosocial outcomes that truly matter to its beneficiaries are captured and converted to monetary terms through stakeholders engagement [24]. Over the past decade, SROI has increasingly been adopted for the evaluation of health interventions, particularly in mental health in areas such as depression, dementia and illegal substance use, and an alcohol recovery program for dependent drinkers [25–29]. However, to our knowledge, a SROI approach has not been used to evaluate any integrated programs dealing with different severity levels of alcohol problems [30].

This study aimed to estimate the impacts and social value, as compared to the costs, of i-MAP in a primary care setting on its relevant stakeholders. It was designed to complement existing evidence through the use of an alternative viewpoint in order to assess whether the provision of such an intervention package offered good value for money.

## Materials and methods

### Study design

We used a mixed-method approach consisting of in-depth stakeholder interviews and two phases of cross-sectional surveys. The study was conducted based on the SROI approach as described elsewhere [31]. This study was approved by Office of Human Research Ethics Committee, Prince of Songkla University (approval number 59-232-18-8).

### Study setting and sample

Study sites were four community hospitals (Singhanakhon, Satingphra, Bangklam and Rattaphum) in Songkhla province of southern Thailand where i-MAP has already been adopted in usual care. Two different groups of participants were recruited between January and April 2017, as this period covered both non-festive and festive seasons (Thai New Year week in mid-April) which differentially influence the population’s drinking patterns. The first group involved in the cost collection and outcomes measurement surveys included consecutive patients aged 15 years and older who received screening and interventions corresponding to their drinking levels. Those with comorbid severe mental illnesses, illegal substance use or profound cognitive impairment were excluded. Of 131 drinkers identified for inclusion, 18 drinkers (all of whom were higher-risk drinkers) declined to enter the program because of “insufficient time”. They received only brief advice, and hence, did not comply with the i-MAP protocols and were excluded from the study. Accordingly, informed consent to participate was obtained from 113 individuals (comprising 29 low-risk, 43 high-risk, and 41 dependent drinkers) thereby yielding a response rate of 88%.

The other group of participants involved in qualitative interviews for outcomes identification included representatives of i-MAP stakeholders (described later in the SROI approach section). They were purposively selected from registration and/or contact lists by nurses and public health volunteers (PHVs). Maximum variation sampling was used in order to construct a robust view from individuals with different backgrounds. Informed consent to participate was obtained from each participant before interview.

### I-MAP protocol at a community hospital

Outpatients were screened at least annually using two questions regarding their recent drinking history (“In your life, have you ever consumed any of alcoholic beverages?” and “In the past 3 months, how often did you usually have at least one drink?”). Those who consumed at least one drink in the past 3 months were subsequently assessed by the Alcohol Use Disorders Identification Test (AUDIT) [32]. Based on the AUDIT results, they were classified into three drinking levels; low-risk (AUDIT < 8) to receive brief education and simple advice to avoid excessive drinking, high-risk (hazardous/harmful; AUDIT 8–19) to receive six sessions over three months of brief interventions (BI) to control risky drinking patterns [33] and probably dependent (AUDIT ≥ 20) to receive medically assisted detoxification followed by six sessions of intensive psychosocial interventions; motivational enhancement therapy (MET) and/or cognitive-behavioral therapy (CBT) designed to reduce addictive behaviors [33]. A full protocol (in Thai) has been published elsewhere [34].

### SROI approach

#### Scope of the study

We projected the 5-year societal outcomes after one-year implementation of i-MAP in a community hospital, as drinking problems tend to relapse over this period [35, 36]. Aggregate costs and benefits of the program were estimated based on the expected annual number of patients with different drinking levels (Table 1).

**Table 1:** Basic assumptions for cost and outcome estimation.

	Value	Source(s)
**Estimated annual number of outpatients, age 15 years and over, per community hospital**	24,573	Primary hospital data (average among 4 setting)
Low-risk drinkers (30%)	7,372	
High-risk drinkers (20%)	4,913	
Dependent drinkers (5%)	1204	
outpatient cases (3.5%)	811	
Admitted cases (1.5%)	393	
**Minimum number of intervention sessions**		Standard guideline [37]
Brief advice/education	1	
Brief intervention	6	
Cognitive behavioral therapy/ motivation enhancement therapy	6	

### Stakeholder identification

Beneficiaries of i-MAP initially included drinkers, families, local communities, healthcare sectors and two other local sectors (police and probation offices). After in-depth interviews, however, the local sectors were subsequently excluded as referral systems for alcohol-related problems had not been implemented in their settings. National-level third sectors (labor market and national legal authorities) were additionally included to represent beneficiaries in the broader society although no primary interview was conducted because we were unable to identify representatives from diverse occupations involved in this stakeholder group.

#### Resources and cost estimation

Resources for the pre-implementation phase (e.g. curriculum design and training) were collected from i-MAP budget reports and presented as average cost per study site. Implementation resources were collected among the outpatient sample using the activity-based approach and presented as average unit(s) used per case. After each visit, key staff completed an activity record form consisting of type and duration of intervention, number and position of staff, medical supplies, waiting time and out-of-pocket expenses. Socio-demographic and drinking-related characteristics of the drinkers were also collected at the first visit. Hospitalisation data were based on individual clinical record forms. Each cost component was estimated by multiplying the unit(s) of resource by corresponding national standard price. Labor and opportunity costs were estimated based on human capital approach by multiplying hours spent on the program by hourly wage. Minimum labor wage was applied for drinkers as they tend to be unemployed or have low productivity [38]. However, population average income was alternatively applied in sensitivity analysis. Overhead costs were estimated apart from cost of routine service delivery. For instance, electricity cost was calculated from the area of the alcohol clinic multiplied by the average electricity cost per hospital utility area.

#### Outcomes identification and evidencing

The list of outcomes was identified through in-depth interviews, conducted by the principal investigator, with representatives of stakeholder groups who had experiences in i-MAP participation. Across all settings, there were four interviews (4–10 participants each) with past i-MAP clients (drinkers), four interviews (3–7 participants each) with families of past clients, three interviews (2–6 participants each) with PHVs and two individual interviews with clinical nurse specialists. Participants described their role in the program and changes, either positive or negative, they experienced after program involvement. Except for the drinker group, other stakeholders (e.g. healthcare stakeholders) were also asked to identify changes to drinking among their respective groups. Each interview, lasting 45–90 minutes, was audio recorded with consent and transcribed by the research assistant. Important outcomes were identified using thematic analysis approach [39].

Most participants in drinker groups reported significant changes in “improved self-esteem”, “better decision-making ability”, and “better emotional control” domains, as a result of self-reflection and positive feedback from the others. These changes for the drinkers were also observed by family members and service providers. “Receive more support” from family and “increased interaction with community” were also common changes, as the drinkers felt that family members were getting closer and the neighbors talked to the drinkers more often as they were not as drunk as before. The drinkers also participated in community event more often as they did not spent most of their time drinking.

The most common changes among families of drinkers included “less argument within family”, “reduced stress” as the mental wellbeing of families improved as they were less worried about the drinkers. “Reduced burden” was also experienced, as they spent less time looking after the drinkers.

Among the other stakeholder groups, public health volunteers reported that “empathetic attitude” toward the drinker was gained as they got more insight on context around drinkers through participation in i-MAP program. Service providers felt that healthcare resources for alcohol-related health conditions and injuries would be saved substantially as less risky drinkers would require less health care services. Also, from a societal perspective, alcohol-related harms such as traffic accidents and productivity losses may be prevented as a result of the program.

Selected outcomes were then used to construct questionnaires for follow-up survey of the outpatient sample and their families at 6 months after completing i-MAP. Drinkers estimated how much they experienced each outcome based on a Likert scale (“obviously”, “somewhat”, “rarely” and “not at all”). Families answered two questionnaires consisting of the outcomes of themselves and their drinking relatives. To minimize over-report, each outcome of the drinker was considered to be achieved only if both the drinker and relative responded with “obviously”. Additionally, AUDIT scores were measured among drinkers to classify post-program drinking status. We assumed that outcomes of healthcare providers and the third sectors (e.g. reduced service use and productivity gain) would result by the move from high-risk/dependent to low-risk drinking.

#### Outcomes valuation

Psychosocial outcomes were monetized using revealed preference techniques, i.e. closest comparable value of products/services with market prices (Table 2). For instance, value of drinker’s ability to better regulate negative emotion would be equal to cost of therapy sessions specifically aiming to improve coping mechanism; value of increased participating in community activity (i.e. increased sense of belonging) would be equal to cost of hiring someone to volunteer in social events. Governmental documents (e.g. service rates in public hospital, minimum labor wage) were set as the first priorities of data sources for all financial proxies in order not to overpricing the outcomes. Governmental documents (e.g. public service rates) were determined as the first basis for data sources in order not to overprice the outcomes. Our analysis was conservative; outcomes of the treatment for low-risk drinkers were excluded because the intervention was too brief to claim any level of effectiveness.

**Table 2:** Outcome indicators, proxy values, deadweights, attributions, durations and drop offs^a^.

Outcomes	Indicators (source)	Financial proxies		Deadweight^b^		Attribution^c^		Duration and drop-off^d^		
		Financial proxies (source)	Value	Rationale (source)	Value	Rationale (source)	Value	Rationale (source)	Duration	Drop off
**Service provider**										
Reduced service use	% of high risk/dependent drinkers improve to abstainer/low risk drinker [20, 40, 41]	Average annual healthcare cost of treatment for all alcohol-attributable medical conditions per high risk/dependent drinker [4]	312 THB*	Proportion of drinkers who spontaneously improved to abstainers/ low risk drinkers [35]	0.25	Proportion of drinkers who reduced/stopped their drinking as a result of other programs e.g. “Quit alcohol during the Buddhist Lent period '' program (drinkers, providers and PHVs interviews)	0.3	Depends on propensity to relapse of excessive drinking: 50% of treated drinkers tend to relapse after 1 year [36]	2 years	0.5^†^
**Drinkers**										
Better decision-making ability	% of drinkers reported better decision / less inappropriate behaviors (follow-up survey)	Cost for behavioral therapy for 6 sessions [42]	3,000 THB	As deficits in judgmental ability is assumed to be fully attributed to high risk drinking, deadweight is equal to rate of high-risk/dependent drinkers spontaneously improved to abstainers/ low-risk drinkers [35]	0.25	As these cognitive deficits is assumed to be fully attributed to high risk drinking, it is impossible for drinkers to be treated with these symptoms without their problem drinking	0	Judgmental ability is largely affected by alcohol hence it depends on propensity to relapse of excessive drinking	2 years	0.5^†^
Better emotional control	% of drinkers reported better emotional stable (follow-up survey)	Counseling fee for stress coping; 6 sessions [42]	1,800 THB	As impulse control disorder is chronic condition and its prevalence is stable overtime, spontaneous remission rate is considered zero [43]	0	Proportion of general population with impaired impulse control receiving any formal treatment during past 1 year (22.8%) [44] and having remission (29–44%) [45]	0.083	Involves changes in coping mechanism, expected to last longer but tend to subside if relapse occurs	5 years	0.5^†^
Improved self esteem	% of drinkers reported improved self esteem	Counseling fee for 6 sessions of supportive psychotherapy [42]	1,800 THB	Remission rate of untreated depression among general population within 1 year [46]	0.53	Proportion of general population with affective disorders receiving any formal treatment during past 1 year [47]	0.16	Involves internal changes of view to oneself, expected to last longer but tend to subside if relapse occurs	5 years	0.5^†^
Receive more support from family	% reported increased positive interaction between drinker and family members (follow-up survey)	Average annual household expenditure for entertainment/ social activities [48]	3,088 THB	Proportion of drinkers’ families reported the pre-existing close relationship between members [49, 50]	0.33	According to providers and public health volunteer interviews, there is currently no identified alternative services/programs contributing this outcome.	0	Depends on propensity to relapse of excessive drinking	2 years	0.5^†^
Increased interaction with community	% report ↑ participation of community activity (follow-up survey)	Volunteer wage for religious activities in 5 major Buddhist days (e.g. Buddhist lent) [51]	1,500 THB	According to group interview, drinkers could not identify what would have happened to their community participation if there was no i-MAP, however they could identify to what extent the outcomes were results of other health promotion programs (see in attribution)	0	Estimated proportion of community participation as results of other health promotion programs e.g. exercise program (drinkers and PHVs interview)	0.3	Depends on propensity to relapse of excessive drinking	2 years	0.5^†^
**Family of drinkers**										
Less argument within family	% of drinkers reported less argument with family (follow-up survey)	Family counseling for >2 persons; 6 sessions [42]	3,000 THB	Proportion of drinkers’ families reported none of pre-existing alcohol-related harm from drinkers [52]	0.63	Estimated proportion of household with alcohol-related domestic violence ever contacted local organization for help (providers and PHVs interview)	0.3	Depends on propensity to relapse of excessive drinking	2 years	0.5^†^
Reduced caregiver stress	% of families reported subjective improved sense of wellbeing	Counseling fee for stress coping; 6 sessions [42]	1,800 THB	Remission rate of untreated depression among general population within 1 year [46]	0.53	Proportion of general population with affective disorders receiving any formal treatment during past 1 year [47]	0.16	Acute change, influence from program was not expected to last longer than a year	1 year	-
Reduced burden	% of families reported increased free time (follow-up survey)	Cost for housekeeping once a month for 1 year	3,600 THB	Proportion of household of drinkers with more than 2 members^e^ [52]	0.71	Estimated proportion of household ever sent problem drinkers to nursing home/halfway house (providers and PHVs interview)	0.1	Depends on propensity to relapse of excessive drinking	2 years	0.5^†^
**Public health volunteers**										
empathetic attitude toward drinkers	% PHVs reported improved basic knowledge of alcohol use disorders and transfer this to their local communities (group interview)	Budget for health promotion/educational program per 1 community [53]	50,000 THB	Proportion of population with at least one drinker in the household^f^ [54]	0.32	Estimated proportion of improved knowledge/attitude toward drinkers as results of other health promotion programs (PHVs interview)	0.3	Involves internal change, a small drop-off was assigned as they may feel burdened	5 years	0.25^ⱡ^
**Third parties**										
Reduced alcohol-related road accidents	% of high risk/dependent drinkers improve to abstainer/low risk drinker	Average cost alcohol-related road accidents (law enforcement, property damage) per high risk/dependent drinker [4]	91.2 THB*	Proportion of high-risk/dependent drinkers who spontaneously improved to abstainers/ low risk drinkers [35]	0.25	Proportion of drinkers who reduced/stopped their drinking as a result of other programs e.g. “Quit alcohol during the Buddhist Lent period '' program (drinkers, providers and PHVs interviews)	0.3	Depends on propensity to relapse of excessive drinking	2 years	0.5^†^
Increase workforce population	% of high risk/dependent drinkers improve to abstainer/low risk drinker	Average alcohol-related productivity loss (premature death, reduced productivity) per high risk/dependent drinker [4]	13,350 THB*	Proportion of high-risk/dependent drinkers who spontaneously improved to abstainers/ low risk drinkers [35]	0.25	Proportion of drinkers who reduced/stopped their drinking as a result of other programs e.g. “Quit alcohol during the Buddhist Lent period '' program (drinkers, providers and PHVs interviews)	0.3	Depends on propensity to relapse of excessive drinking	2 years	0.5^†^

* inflated from 2006 to 2017 value, THB = Thai baht

a: Displacement values were set at zero for all outcomes as i-MAP is proposed to set on top of usual service hence its outcomes would not be supposed to displace other services. Additionally, drinkers are usually disadvantage from social inclusion hence getting treatment would increase social activeness and hardly displace their usual activities.

b: Deadweights were considered on what would be happened if drinkers visiting hospital had been not screened for drinking problem. Prospective cohort and epidemiologic studies were used as sources to estimate natural course of those who did not receive interventions.

c: Depends on nature of the change itself as well as characteristics of its beneficiary. Changes that were associated with behavioral modification and acquired skills and/or were direct result of the program were assumed to last longer, while changes which occurred instantaneously or were indirect effect of the program would last shorter.

d: Rate per year. Changes that were associated with behavioral modification and acquired skills and/or were the direct result of the program were assumed to last longer, while changes which occurred immediately or were indirect effect of the program would last shorter. Each outcome tends to subside as a result of drinking relapse, habituation or lessened influence by intervention itself over time.

e: In a household with two or more non-drinkers, a primary caregiver of drinker has the option to have free time by asking another caregiver(s) to replace him/her.

f: Those who have drinking relatives in their household would, to some extent, have a better understanding of the situation/problems/suffering surrounding drinkers even though the program had not existed.

^†^According to stakeholder interviews, some of participants stated that these outcomes might diminish as soon as the drinking problems recur, Drop off of 0.5 were then assigned based on annual relapse rate of drinking problems for conservative assumption.

^ⱡ^ According to PHVs interviews, they stated that they partly felt tired/ burdened when treatments for drinkers were not successful. Drop off of 0.25 was then assigned for a conservative assumption.

Financial proxy of reduced service use was estimated using the national report on social cost of alcohol consumption in fiscal year 2006 [4]. The report estimated amount of costs of diseases, injuries, crimes and productivity losses directly related to drinking by application of corresponding alcohol-attributable fractions (AAFs). We calculated avoided healthcare cost per case by dividing annual alcohol-related healthcare cost by the estimated number of high-risk drinkers (in 2006) as we assumed that the hazardous/harmful consumption would result in adverse events. Financial proxies of avoided crimes and productivity losses were also estimated by the similar calculation. To comply with study timeframe, only acute (e.g. hepatitis and injuries) but not chronic consequences (e.g. cancers and cirrhosis) were included in our analysis.

#### Impact calculation

To ensure the credibility of the results, the following factors influencing the impact of the program needed to be considered; *deadweight* (proportion of outcome that would have happened anyway even if the program had not existed, equivalent to “no treatment/ treatment as usual” scenario in traditional economic evaluations), *attribution* (proportion of the outcome that could be attributable to other programs), *displacement* (proportion of the outcome that could displace other programs), *duration* (how long the outcome would last) and *drop off* (proportion of the outcome expected to diminish in the next year). The sources of these values were obtained from published observational and experimental studies and/or stakeholder interviews. The general and specific rationale for each assigned value is described in Table 3.

**Table 3:** Basic characteristics of sample and inputs of i-MAP Health program by drinking status.

Baseline characteristics	Value			
	Low-risk (n = 29)	High-risk (n = 43)	Dependence (n = 41)	Total
**Mean age, mean (95%CI)**	40.5 (35.7, 45.1)	47.9 (44.6, 51.1)	47.5 (44.6, 50.4)	46.0 (43.9, 48.1)
**Age group (%)**				
20–29	18.5	7.0	0	7.2
30–39	25.9	11.6	22.0	18.9
40–49	37.0	32.6	41.5	36.9
50–59	11.1	32.6	22.0	23.4
60+	7.4	16.3	14.6	13.5
**Male: female, proportion**	100:0	100:0	97.6:2.4	99.1:0.9
**Past year legal involvement (%)**				
**None**	85.2	76.7	68.3	75.7
1 event	11.1	18.6	14.6	15.3
2+ events	3.7	4.7	17.1	9.0
**Current employment rate, (%)**	81.5	88.4	73.1	81.1
**Accompanied by relative(s), (%)**	29.6	30.2	65.9	43.2
**Time spent by activity, (min.)**				
Screening	16.2 (11.0, 21.5)	18.7 (14.5, 22.6)	22.3 (16.6, 28.0)	19.4 (15.5, 22.4)
Intervention	19.6 (15.0, 24.1)	38.1 (30.6, 45.3)	42.2 (36.6, 48.3)	36.9 (32.6, 41.0)
**Time spent by professionals (min), mean (95%CI)**				
Nurse; 1^st^ visit	37.4 (28.52, 46.30)	56.8 (47.5, 66.2)	64.5(55.9, 73.0)	56.6 (50.6, 62.2)
Nurse; next visits	-	38.1 (30.6, 45.3)	42.2 (36.6, 48.3)	36.9 (32.6, 41.0)
Physician	11.6 (5.3, 17.9)	13.0 (9.0, 17.3)	12.3 (8.0, 16.5)	12.4 (9.8, 15.3)
Pharmacist	5.6 (3.6, 7.7)	8.4 (5.3, 11.4)	8.5 (6.5, 10.6)	7.8 (6.3, 9.2)
**Length of hospital stay (days), mean (95%CI)**	-	-	3.8 (2.5,5.0)^a^	
**Medications (% received)**				
Benzodiazepines	25.9	46.5	87.8	56.8
Antipsychotics	0	14.0	31.7	17.1
Supplements	22.2	39.5	85.4	52.3
**Lab test (% received)**				
CBC	3.7	18.6	46.3	25.2
Blood glucose	11.1	9.3	39.0	20.7
Electrolytes	7.4	7.0	48.8	22.5
Liver function test	14.8	23.3	51.2	31.1
BUN	18.5	25.6	48.8	32.4
Creatinine	18.5	16.3	41.5	26.1
**Patient resources, mean (95%CI)**				
lost time (min)	190.6 (154.6,226.0)	174.7 (152.6, 197.5)	197.1 (176.8, 218.8)	186.8 (171.5, 201.8)
Out of pockets /visit (THB)	76.8 (65.5, 88.2)	76.8 (65.5, 88.2)	76.8 (65.5, 88.2)	76.8 (65.5, 88.2)

a; mean among those who were hospitalised (n = 12), CI: confidence interval, CBC: complete blood count, BUN: Blood Urea Nitrogen, THB: Thai baht

#### SROI ratio calculation

The total investment was sum of annual costs of management protocols for all drinking levels. Before calculating the total return, the value created in each successive year after implementation was calculated by summing all benefits incurred in that year adjusted by impact influencing factors, expressed as;

$$\mathrm{Return}\;\mathrm{in}\;\mathrm{year}\;(j)=\sum_{i}^{n}{E_{i}B}_{i}[(1-D_{i})(1-A_{i})(1-P_{i}){\left(1-O_{i})\right]}^{j}$$

Whereby n is number of selected outcomes. E_i_ and B_i_ are quantity and proxy value of achieved outcome(i), respectively. D_i_, A_i_, P_i_ and O_i_ denote deadweight, attribution, displacement and drop off of outcome(i), respectively, at year(j). It should be noted that outcomes that last only for (j) year were excluded from calculation of year(j)+1 and later.

In addition, return in each future year was expressed in relation to current value (present value; PV), using a constant discount rate (r) as the following expression.

$$\mathrm{PV}\;\mathrm{of}\;\mathrm{total}\;\mathrm{return}=\sum_{j}^{n}total\;return\;of\;year\;(j)/{\left(1+r\right)}^{j}$$

A discount rate at 3% was used in base case analysis [55]. Ultimately, SROI was expressed as total adjusted return divided by total investment. The ratio that is greater than 1 implies that the return of the program outweighs the costs, indicating worthwhile of investment.

#### Statistical analysis

Socio-demographic and drinking-related characteristics of participants in the quantitative part were analyzed using R. Categorical and continuous data were presented in percentages with standard errors (s.e.) and means with 95% confidence intervals (95%CIs), respectively. Due to positive-skewed distribution, 95%CI of cost data were generated using a bootstrap method [56]. Sources for costs reported in previous years were inflated to the present (2017) using the consumer price index [57].

#### Sensitivity analysis

In one-way sensitivity analysis, the following parameters were tested individually; *discount rate* at 0% and 6% [4], *Quantity of outcomes* increased/decreased by 20%, *deadweights* and *attributions* increased/decreased by 20%, shorter *timeframe* of 1 year, *opportunity cost estimation* using national average income and application of *unemployment rate* among drinkers, derived from a national report [52] and our survey, to adjust opportunity costs and productivity gains.

Additionally, probabilistic sensitivity analyses of high-risk and dependent drinkers were separately performed using Monte Carlo simulations in Excel. Key variables included cost components and probabilities of achieved outcomes which were randomly drawn 1,000 times based on gamma and beta distributions, respectively [58].

## Results

Participants in the quantitative portion of the study were almost exclusively male with the mean age of 46 years. In all drinking subgroups the highest prevalence occurred in the age range 40–49 years. Drinkers in higher risk groups were more likely to be involved in legal actions, require more staff time, medical supplies and laboratory tests. However, they were not different regarding waiting time (Table 3). All participants received the minimal set of intervention sessions according to their initial AUDIT score. The 6-month follow-up survey was completed by 95% and 86% of high-risk and dependent drinkers (and their primary caregivers), respectively.

Total annual cost of i-MAP in a community hospital was estimated at 25,500,000 baht (US$815,000), of which approximately 41% was constituted by labor cost and 26% by opportunity cost of patients. Disaggregated by drinking status, almost 60% (14,800,000 baht or US$473,000) of total cost pertained to high-risk drinkers, followed dependent (6,700,000 baht or US$214,000) and low-risk drinkers (4,300,000 baht or US$139,000). Average implementation cost per low-risk, high-risk, dependent drinker who was not hospitalised, and dependent drinker who was hospitalised were 516 (US$16), 2,961 (US$94), 3,810 (US$120) and 9,861 (US$310) baht, respectively.

Total adjusted value predicted to be created at 5 years after implementation was approximately 51,000,000 baht (US$1,600,000), of which two-thirds would incur by the first year. Half of total value was generated to broader society, followed by drinkers (37%) and families (9.6%). In contrast, value created to healthcare sector accounted for less than 1% of total value (Table 4).

**Table 4:** Outcome indicators, proportion and estimated quantity of stakeholders achieved indicators and adjusted present values.

	Proportion achieved indicator (s.e.)		Estimated number of stakeholder	Present values†					
	High-risk (n = 41)	Dependence (n = 37)		1^st^ year	2^nd^ year	3^rd^ year	4^th^ year	5^th^ year	Total
**Service provider**									
Reduced service use	0.42 (0.07)	0.46 (0.08)	2,616	416,020	201,951	-	-	-	617,972
**Drinkers**									
Better decision-making ability	0.24 (0.07)	0.24 (0.07)	1,468	3,206,971	1,556,782	-	-	-	4,763,753
Better emotional control	0.27 (0.07)	0.24 (0.07)	1,615	2,588,830	1,256,714	610,055	296,143	143,759	4,895,501
Improved self esteem	0.29 (0.07)	0.08 (0.04)	1,521	1,049,463	509,448	247,305	120,051	58,277	1,984,545
Receive more support from family	0.27 (0.07)	0.22 (0.07)	1,591	3,196,624	1,551,759	-	-	-	4,748,383
Increased interaction with community	0.17 (0.06)	0.03 (0.03)	871	1,550,756	752,794	-	-	-	2,303,550
**Family of drinkers**									
Less argument within family	0.34 (0.07)	0.32 (0.08)	2,056	888,249	431,189	-	-	-	1,319,438
Reduced caregiver stress	0.49 (0.08)	0.49 (0.08)	2,997	2,067,983					2,067,983
Reduced burden	0.17 (0.06)	0.22 (0.07)	1,100	1,003,538	487,155	-	-	-	1,490,693
**Public health volunteers**									
empathetic attitude toward drinkers	NA	NA	1*	23,107	16,825	12,251	8,921	6,496	67,600
**Third parties**									
Reduced alcohol-related road accidents	0.42 (0.07)	0.46 (0.08)	2,616	121,606	59,032	-	-	-	180,638
Increase workforce population	0.42 (0.07)	0.46 (0.08)	2,616	17,800,857	8,641,193	-	-	-	26,442,050
**Total**				**33,914,005**	**15,464,842**	**869,612**	**425,115**	**208,532**	**50,882,105**

*Unit of achieved outcome indicator is number of district.

† in Thai baht, adjusted with 3% discount rate. s.e.: standard error.

**Fig 1:**
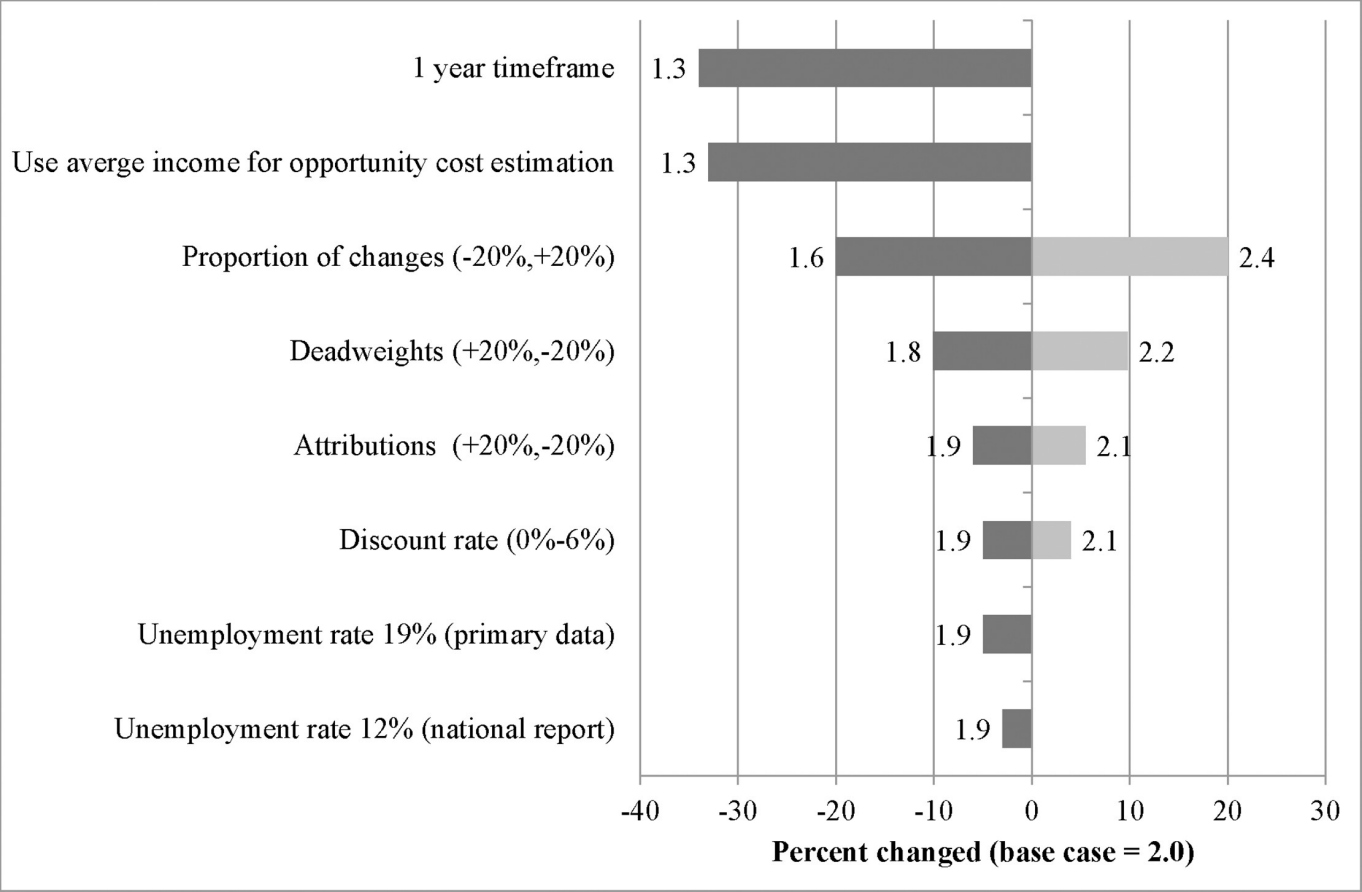
Tornado plot of one-way sensitivity analysis.

Accordingly, SROI ratio generated by i-MAP was 2:1. One-way sensitivity analyses show that the alternative ratios ranged from 1.3 to 2.4 (Fig 1). Using alternative timeframe and source for opportunity cost both decreased the ratio by one-third. Adjusting proportions of achieved outcomes changed the ratio by 20%. In contrast, changing discount rate and applying unemployment rates did not significantly alter SROI ratio.

**Fig 2:**
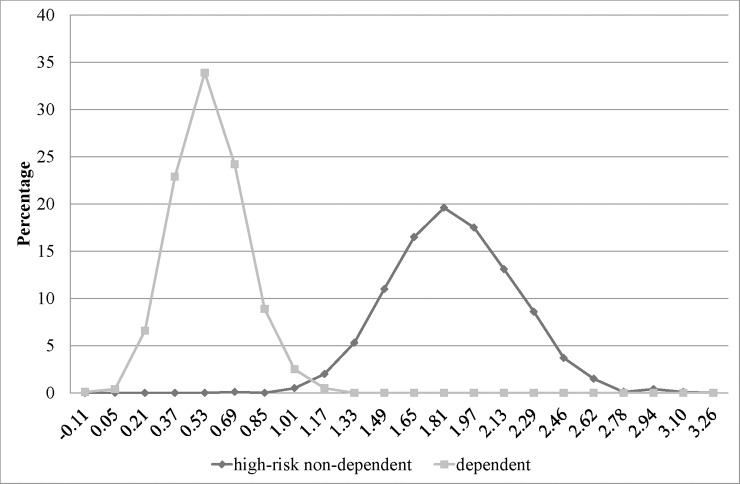
Line graphs illustrating probability distribution of net SROI of treatments for high-risk and dependent drinkers.

**Fig 3:**
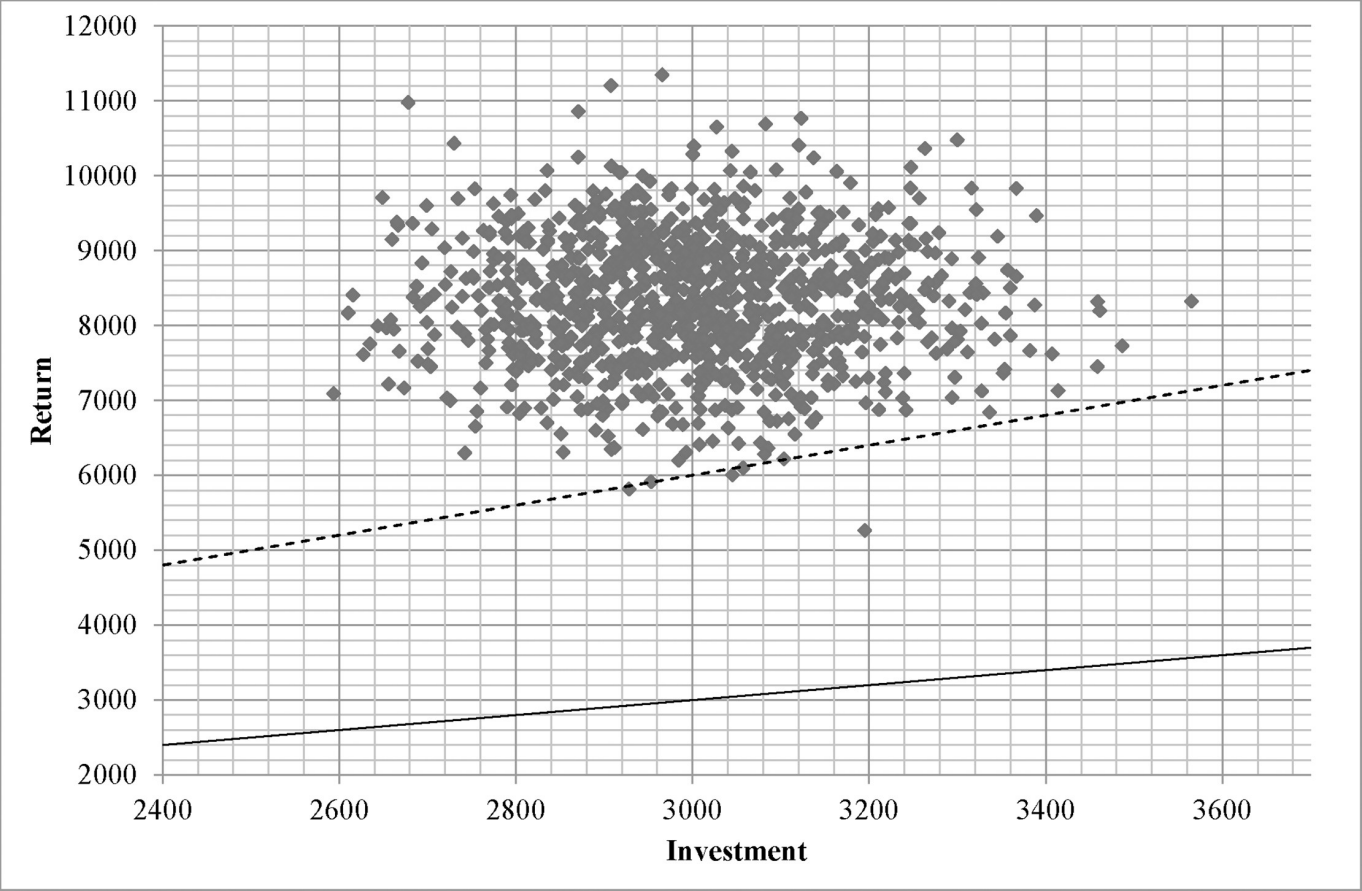
Scatter plot representing 1,000 Monte Carlo simulations of costs and returns of treatments for high-risk drinkers. Dash lines define neutral net SROI lines (net SROI ratio = 1) and solid lines define neutral SROI lines. (SROI ratio = 1).

**Fig 4:**
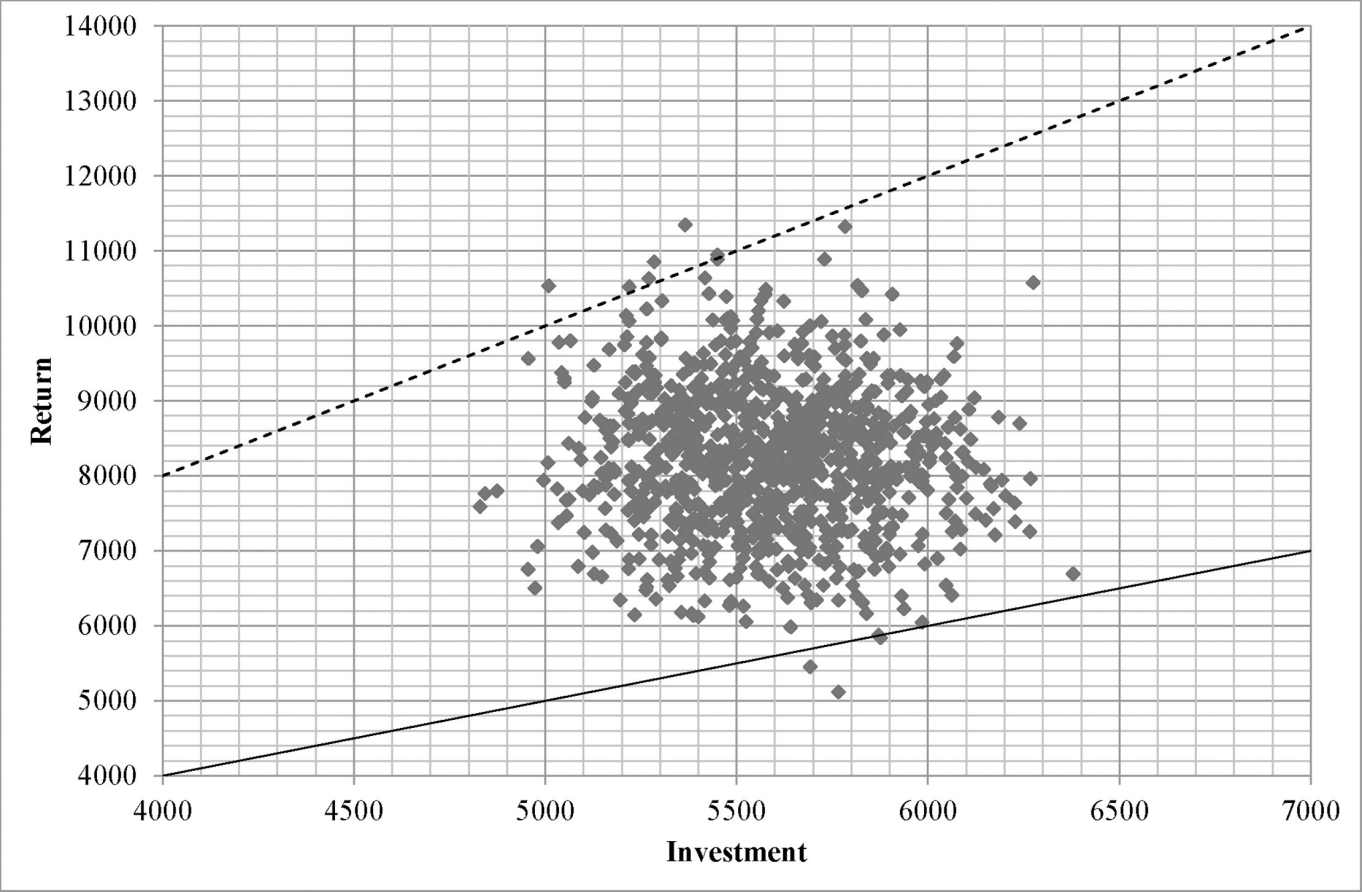
Scatter plot representing 1,000 Monte Carlo simulations of costs and returns of treatments for dependent drinkers. Dash lines define neutral net SROI lines (net SROI ratio = 1) and solid lines define neutral SROI lines. (SROI ratio = 1).

SROI ratio of treatment for high-risk drinkers was around twofold greater than that for dependent drinkers (2.8 vs. 1.5), with approximate ranges of 2.0 to 3.2 and 1.2 to 2.0, respectively (Fig 2). Fig 3 and Fig 4 illustrate relationship between cost and return of treatment for each drinking group. All and 99.7% of simulated returns of treatment for high-risk and dependent drinkers, respectively, were greater than the corresponding costs.

## Discussion

This study shows that implementation of the i-MAP program in a community hospital yields net positive value to drinkers, their families, healthcare sector, local community and broader society. The SROI ratio ranged from 1.3 to 2.4 baht for every baht invested, though it was relatively low compared to that of previous cost-benefit studies of alcohol interventions [21]. The reason may be that some of the previous studies examined among those with severe conditions, sequelae of which incurred a great loss, hence even little improvement produced great benefit. Another reason may be difference in value assignment for benefits. For instance, Fleming et al found that brief physician advice produced very large societal benefit-cost ratio of 39:1 [17]. In contrast to our study, they counted all outcome events without taking any AAF into account, making healthcare cost saving very high. In our study, AAFs were applied to generate healthcare/crime cost savings from alcohol interventions. As can be seen, either partly attributed assignment underestimates or fully attributed assignment overestimates the result.

Also our ratio was in the lower range as compared to both international and domestic SROI analyses on other health programs [30, 59]. This may be because studies with greatest SROI ratios mostly evaluated health promotion campaigns which often involve fixed costs rather than variable costs, as opposed to treatment programs, so that budgets were relatively small and widespread benefits were produced. Although prevention is generally better than cure, some individuals may still need some more effort for their changes to occur. For certain alcohol users, screening and interventions at hospital may be their only “teachable moment” as physical complaints may be only obvious problems related to their underlying drinking.

Most of benefits of i-MAP pertained to labor market and drinkers. Productivity losses from sickness absenteeism and presenteeism (working while ill), and premature mortality were found to represented majority of measurable costs attributable to alcohol [3, 4]. Consequently, each problem drinker whose consumption reduces or stops as a result of intervention would substantially avoid these losses. Of all outcomes identified by the drinkers, receiving support, better decision-making and emotional control were most valuable changes created because of being most common responses and lower attributions and deadweights. According to qualitative interviews, families often kept their distance and avoided confronting the drinkers either because of being frightened or tired of them. Receiving treatment created more positive attitude towards the drinkers and, consequently, supportive interactions within families. Adaptive life skills were better developed through empathetic and supportive treatment environment as a result of either improvement in drinking behaviors or premorbid personality per se [22, 60, 61]. Regarding the local community perspective, “empathetic attitudes…” was only identified change among PHVs. However, the attitude in fact could be transferred to the wider community hence benefit to this group could be underestimated.

Although we excluded benefits to low-risk drinkers to avoid overclaiming, the program might contribute benefits to them to some extent. Considering that some of them might in fact be former high-risk drinkers, the program could assist or maintain their safe drinking. Interestingly, even screening and assessment per se were found to trigger behavioral modification to some extent [14, 62].

Sensitivity analyses show that the most variant parameters pertain to alternative opportunity cost estimation and intervention effectiveness. SROI ratio of high-risk drinking treatment was greater than that of dependence treatment. This is not surprising considering that dependent users are more difficult to treat. In other words, our result supports that early prevention of alcohol problems is worth more than late intervention [21]. It should be noted that, however, benefit of alcohol dependence treatment might be underestimated as cost-savings from long-term consequences were excluded in our analyses [63].

Unexpected findings that 26% of low risk drinkers and 47% of high risk received benzodiazepines, while 14% of high risk patients received anti-psychotics, seem surprisingly high, as most of these individuals would not need treatment for alcohol withdrawal. As we obtained data from clinical record forms retrospectively, it would be difficult to obtain a precise explanation for the administration of benzodiazepines/antipsychotic drugs (usually by GPs) for each individual case. Possible explanation, after interviewing attending nurses at the study sites, may be that some of these high-risk drinkers were known to have a prior history of heavy drinking or withdrawal syndrome so that the physicians might prescribe those drugs to prevent their recurrence. Nevertheless, our unreported data show that most of the medicines prescribed to these drinkers were at low dosage (e.g. 2 mg per day of haloperidol).

### Strengths and limitations

To our knowledge, this is the first economic study evaluating an alcohol program as a whole package in which sets of interventions were flexibly delivered to various groups of drinkers [21]. As alcohol users are different in their needs and preferences, adhering only to a single treatment regimen, as in randomized controlled trials, may not suitable in real-life practice [64] and health services should rather provide comprehensive intervention for alcohol users with different severities [65]. Our findings add knowledge, by considering all relevant beneficiaries, on what and how much social values would be created from integration of such a program package into primary care.

Nevertheless, it should be noted that the SROI approach is rather a broad concept, without a commonly accepted method for financial valuation of the benefits. Our results rested on the assumption that all outcomes could be monetized using a financial proxy. Although various techniques had been used to estimate willingness-to-pay for intangible outcomes related to alcohol, such as harm, productivity loss and quality of life [66, 67], such estimations are also subject to overestimation and highly varied, depending on a number of internal and external factors [68]. For instance, use of the cost of providing therapy (e.g. counseling for stress coping) as a proxy for stress reduction when a person reduces or stops drinking is likely to inflate or reduce the real cost of reducing stress. The benefit of reducing harms attributed to hazardous drinkers may be slightly over-estimated in this analysis as a result of the assumption that all risky drinkers (those with AUDIT of 8 or more) could experience harms. A comparative study on different valuation approaches particularly for alcohol-related treatment outcomes conducted in the same context would contribute a substantial insight into these issues.

We forecast SROI based on a situation when i-MAP had been perfectly implemented and the target population was accurately assessed and completely attended the program. Resource constraints, e.g. professional workforce, concurrent burden and limited mental energy of staff were not taken into account. Moreover, some drinkers might in fact underreport their problems, refuse or fail to complete the program. Further studies thus should be conducted with these real-life constraints taken into account. In spite of that, this study could inform the policymakers regarding how much resources could be allocated to maximize value of the program. Due to study constraints such as time limit and difficulty in case finding, an almost exclusively male sample could limit generalizability, although this may actually reflect the high gender disproportion in the nation’s alcohol problems [47]. Also selection bias might arise as participants were those who wanted to change their behaviors. However, considering that those who were not ready to change had no chance to receive benefits from the program, this may not significantly impact the SROI ratio.

## Conclusions

As with other mental health problems, capturing only drinking-related outcomes could obscure the true merit of alcohol interventions. This study, by using a SROI approach, demonstrates that the benefits could be over twice the investment costs of integrating the i-MAP program into primary care. The finding that treatment for non-dependent and dependent drinking yields positive net benefits, though the latter is more costly, could further support the application of alcohol interventions to all types of alcohol users in clinical practices.

## Supporting information

S1 File Dataset. (CSV)
